# Immune Dysfunctlon in Patients With Obstructive Jaundice, Mediators and Implications for Treatments

**DOI:** 10.1155/1997/49076

**Published:** 1997

**Authors:** W. G. Jiang, M. C. A. Puntis

**Affiliations:** University Department of Surgery University of Wales College of Medicine Heath Part Cardiff CF4 4XN UK

## Abstract

Patients with obstructive jaundice have an increased perioperative complication rate. Sepsis, bleeding, wound problems, renal and liver malfunction are all seen in these patients. Assessment of immune function has been an active research area in these patients. This review will examine various aspects of immune functions in obstructive jaundice, discuss the recent research results and controversies and then go on to discuss the relevant mediators of immune function and some possible implications for treatment.


*HPB Surgery*, 1997, Vol.10, pp.129–142
Reprints available directly from the publisher
Photocopying permitted by license only

© 1997 OPA (Overseas Publishers Association)
Amsterdam B.V. Published in The Netherlands
by Harwood Academic Publishers
Printed in India

### Immune Dysfunction in Patients with Obstructive Jaundice, Mediators and Implications for Treatments

W.G. JIANG and M.C.A. PUNTIS

University Department of Surgery, University of Wales College of Medicine, Heath Part, Cardiff CF4 4XN, UK

*(Received 1 March 1996)*

Patients with obstructive jaundice have an increased perioperative complication rate. Sepsis, bleeding, wound problems, renal and liver malfunction are all seen in these patients. Assessment of immune function has been an active research area in these patients. This review will examine various aspects of immune functions in obstructive jaundice, discuss the recent research results and controversies and then go on to discuss the relevant mediators of immune function and some possible implications for treatment.

KEYWORDS: Obstructive jaundice immune function cytokines

It is accepted that patients with obstructive jaundice have increased perioperative complications and mortality[1]. Collected data has shown that the mortality can be as high as 13%, particularly in patients with: haematocrit less than 30%, bilirubin higher than 200μm/l, and with malignant disease[2]. The common complications are: bacteribilia and sepsis[3–8], gastrointestinal bleeding[1,5,7,9], gastroduodenal mucosal erosions[10], renal failure[1,11,15], impaired wound healing[1,6,7,16], haemorrhage and blood coagulation disorders[5,8,17] including disseminated intravascular coagulation (DIC)[9,8]. This group of patients is therefore of great interest and importance in surgical practice and research.

### IMMUNE FUNCTIONS IN OBSTRUCTIVE JAUNDICE

### *Reticuloendothelial System (RES)-Mononuclear Phagocyte System*

Historically, the term RES was used to describe a population of cells in the reticular connective tissue of the spleen, liver and lymphoid tissue. The alternative Mononuclear Phagocyte System is sometimes used nowadays. This population includes: monocytes, mature macrophages, histiocytes in the tissues, alveolar macrophage in the lung, Kupffer cells in the liver, and macrophages in the spleen, lymph nodes, peritoneum, and other areas.

These cells play a central role in immune regulation[19]. Apart from being phagocytic and antigen presenting, they constitute a secretory organ producing over 100 substances[20]. These range from molecules as small as 32 kDa up to 440 kDa and include: a variety of lytic enzymes, proteins, lipid metabolites and oxygen free radicals. In recent years the monocyte/macrophage has been found to be a principal source of TNFα, IL-1, IL-6, IL-8, and macrophage inflammatory proteins (MIPs)[20,21]. It is also a major contributor of other cytokines: GM-CSF, M-CSF, EGF, IGF-I, IL-10, IL-12, TGFβ PDGF and IFN[21–26].

Much of the work on jaundiced patients has concentrated on the RES phagocytic function. For many years, it has been recognised that the RES phagocytic and clearance functions are disturbed in jaundice. In 1957 Halpern[27] showed that in bile duct ligated rats the RES phagocytic activity for carbon particles was stimulated, later work produced contradictory results. Table I summarises the RES functions in both animal and human studies.

Other aspects of the monocytic system have been studied to a lesser extent. Lee[34] showed that in

TABLE I RES functions in jaundice

| Species | Assay | Comment | References |
|---|---|---|---|
| Rats | Carbon infusion | Phagocytosis-increased | 27 |
| | Sulphur colloid | Uptake-decreased | 4, 28 |
| | Bacteria | Clearance-delayed | 29 |
| | Staphylococcus aureus | Lung macrophage phagocytosis-decreased | 30 |
| | Endotoxin | Kupffer cell uptake-increased | 31 |
| | Bacteria | Kupffer cell uptake-decreased | 32 |
| | Candida albicans | Kupffer cell uptake-decreased | 33 |
| | Cell Migration | RES cell-movement reduced | 34 |
| | Bacteria | Clearance-decreased | 35–39 |
| | Endotoxin | Kupffer cell clearance decreased | 40 |
| Dogs | Bacteria | Liver uptake no-change | 41 |
| Human | Microspheres | Clearance-decreased | 42 |
| | Microaggreated albumin | Clearance-decreased | 43, 44, 45 |

jaundiced rats, the migration of reticulo-endothelial cells and fibroblasts into granulomata was impaired and this may contribute to the delay in wound healing observed in jaundice. Hultberg in 1981[48] reported that patients with jaundice have a markedly increased blood level of one of the lysosomal hydrolases, acetyl-β hexosaminidase (EC 3.2.1 52), an enzyme, which is critically deficient in Tay-Sachs and Sandoff syndrome[49,50]. An increased plasma enzyme level is seen in sepsis[51], necrotizing enterocolitis[52] and some malignancies[53–55]. Hultberg and others[48,56,69] suggested that this may be due to decreased clearance by Kupffer cells in these diseases. Besides enzyme clearance the monocyte/macrophage is also a rich source of this enzyme, secreting lysosomal enzymes spontaneously[57] and in response to stimuli[58,59]. This has lead to the study of the relationship between monocyte/macrophage hexosaminidase secretion and the plasma hexosamindase levels in these patients. It appears that monocytes from these patients have increased production of hexosaminidase both spontaneously and after stimulation[60]. These cells also have an increased total enzyme content, suggesting that increased production from activated monocyte/macrophages, as well as decreased clearance, may both contribute to the increased blood level of hexosaminidase.

Although most studies have shown impaired RES function in jaundice, some recent studies, have, however, shown the converse. Adachi *et al.*[31] in rat studies, have shown that the multiple functions of hepatic macrophages may be separately modified: the phagocytic index for injected $^{51}$Cr-endotoxin is increased 2 weeks after ligation of the bile duct. Superoxide and prostaglandin E2 production by hepatic macrophages and peripheral monocytes are also increased, but IL-1 release is not changed and IL-2 receptors on hepatic macrophages are reduced. This indicates that macrophages in jaundiced animals demonstrate altered function but is this "malfunction"? Allen *et al.*[41], directly measured the hepatic uptake of bacteria in a dog model and found there was no difference in bacterial uptake between controls, partial or complete obstructive jaundice. Semeraro[46] showed that jaundiced patients' monocytes have a greatly increased procoagulant activity (tissue factor) and this may contribute to activation of blood coagulation in severe jaundice. A recent study from Hines *et al.*[47] has shown that after bile duct ligation, liver macrophages show a significantly increased proliferation and activation, which last up to 4 weeks.

Assessment of RES function in intact animals is problematic, because although the mononuclear phagocytic system is one of the key factors in the clearance of bacteria and particles from the blood stream, the process of clearance is very complex and the RES is only part of the process. Various opsonins: (such as IgG, IgM, complement factors, and adhesion molecules), receptors, intracellular signalling pathways, and the cytoskeleton are also required. An *in vivo* clearance study of infused particles (bacteria and others) is therefore not able to define the specific defect in the clearance process[61]. There are similar problems with any evaluation of the restoration of the RES phagocytosis defect. The stimuli used in most studies are not specific for phagocytosis, or even for the RES itself. They almost all stimulate other functions of the RES (superoxide production for example) and other immune functions. More specific and precise methods are needed to define the multiple aspects of RES function in these patients.

*Neutrophils*

Neutrophils, the largest population of immune cells, play a key role in destroying infecting micro-organisms *in vivo*. These cells are normally "at rest" but can be stimulated to make a response, for example, antibacterial function. This will involve chemotaxis, phagocytosis, intracellular killing (by activation of a non-mitochondrial oxidase system) and secretion of various products. Recently, another neutrophil functional state, known as "primed" has been recognised [62,63]. In this state, neutrophils are "prepared for action" and respond to stimulation in an aggressive hyper-reactive manner. This is not merely an additive or synergisitic effect of activating agonists[64,65] but results from an as yet unidentified chemical change within the neutrophils.

*Chemotaxis an d Phagocytosis*

Neutrophil phagocytosis of *S. Aureus* is reduced in jaundiced patients, but the phagocytosis of zymozan is intact[30,66]. Reduced phagocytosis may have some bearing on the morbidity and mortality of jaundiced patients[67]. The chemotaxis of neutrophils has been shown to be impaired[68], but this has not been confirmed by others[30,66].

*Superoxide Production and Priming by Cytokines*

In biliary obstruction increased superoxide production by neutrophils, after stimulation with fmlp, PMA or zymozan can last as long as 15 days[83]. This has been seen in both jaundiced animals[86] and in patients [87]. It has been shown that jaundiced patients with a poor immediate clinical outcome have higher neutrophil superoxide production prior to death[88]. Neutrophils from jaundiced patients, unlike controls, cannot be primed by cytokines TNFα, IL-6, or IL-8[87]. Culture of normal neutrophils with jaundiced serum fails to reproduce the increase of superoxide production observed in jaundice. This may indicate that other elements may be required, for example monocyte/macrophages and the cytokines they can produce[83]. In these patients there is an increased blood level of such cytokines and an increased production from monocytes. It has been suggested that neutrophils from jaundiced patients are 'pre-primed' *in vivo* and therefore produce more toxic products when subjected to subsequent stimulation. Some of the bile salts, which are markedly increased in jaundice may also prime neutrophils at low concentration and stimulate neutrophil at higher concentrations and this may exaggerate the production of superoxide in jaundice[89].

Schmeling[90] reported that PMA when injected into bile ducts results in neutrophil infiltration into the duct tissue and subsequent duct necrosis and fibrosis. This suggests a potential role for neutrophils in the pathogenesis of tissue damage in jaundice. Neutrophil adhesion, an important prelude to extravasation is also impaired in jaundiced[91].

The ability of neutrophils to be primed is interesting. Various cytokines (IL-1, TNFα, IL-6 etc) and interestingly, LPS and some bile salts are able to prime neutrophils at low concentrations. The increased cytokine levels, endotoxaemia and bile salt levels in jaundiced patients may therefore be of particular importance. Various primers can act together to give synergistic effects on neutrophil functions[92]. Non-cytokine primers also include leukotriene B4, cathepsin G, elastase, ATP, lithocholate[89], LPS[62,93] and inositol 6 phosphatase (InsP6). It is important that these various primers can enhance the production of reactive oxygen metabolites, (such as $H_2O_2$, hypochlorite and superoxide) and proteolytic enzymes (such as collagenases and elastases), which all have a potential for causing tissue damage. There is both clinical[94-97, 101-103] and experimental[98-100] evidence suggesting that:

(1) tissue damage from activated neutrophils, including damage to endothelial cells, lung, heart, kidney, cartilage and liver, is greatly enhanced by "priming",

(2) the increased concentration of circulating primers, neutrophil priming and the patients prognosis are all closely linked.

In summary, the priming of neutrophils is a crucial part in the beneficial anti-microbial activity of these cells, but it may increase the harmful generation of tissue damaging molecules. When the systemic levels of "priming" agents reach a critical point, the damage caused by activated neutrophils may trigger a feedback loop in which further inflammation, cytokine production and tissue damage can eventually lead to multi-organ damage. As more and more primers are discovered, it is becoming clear that strategies aimed at preventing the action of an individual agent may be futile. There remains a need to find effective, safe and economic strategies for controlling neutrophil priming.

### *Lymphocytes*

Although lymphocytes are one of the most important parts of the immune system, the changes in this population have been rather less well studied in jaundiced patients.

Vierucci[104] reported that patients with cholestasis have a decreased lymphocyte rosette formation (E rossettes) which is reversible by culture with normal human sera. There is an inhibition of cellular immunity in jaundice and this may be due to endotoxin[37,105].

It has been shown in animal studies that obstructive jaundice inhibits the lymphocyte stimulation index using PHA, conconavalin A or pokeweed mitogen[106,107]. This may be due to agents in the jaundiced blood as jaundiced lymphocytes when cultured with control serum have their impaired function restored. It has also been shown that patients with jaundice, particularly those with bilirubin over 220 μm/l have an impaired delayed hypersensitivity response compared with controls[109]. These results however have failed to be repeated in other studies, in which the T and B cell numbers, T cell responses to mitogens, NK cell functions and serum immunoglobulin were found to be normal[108].

The *in vivo* and *in vitro* mitogenic response of lymphocytes to PHA, allogeneic F344 antigen and concanavalin A are depressed in jaundiced animal models[110–112]. Lymph node cells show similar changes. This suppression can be seen as early as 3 days after bile duct ligation and bears no relationship to the level of bilirubin[113,114].

Finally, IL-2 receptor, an indicator of T cell activation has been found to be increased in patients with obstructive jaundice[115].

### *Humoral Immunity*

Patients with obstructive jaundice of either benign or malignant aetiology have increased levels of secretory IgA, total IgA, and IgA-CIC that correlate with increased C3 levels[116]. These patients also show raised levels of circulating IgA and IgG-containing immune complexes that are correlated with the levles of endotoxin and with bacteraemia[117]. Increased deposition of glomerular IgA is seen in a large proportion of jaundiced patients and in animal models[118,119,120]. These reports suggest a contribution of immune dysfunction to renal failure. Other studies show an increase in CA19-9 [121]. The B lymphocyte functions has been shown not to be affected[110].

### *Cytokines*

Cytokines are a group of protein molecules that are widely produced and are key mediators in many physiological and pathological processes, including immune regulatory effects. For example, TNF, IL-1 and IL-6 are key mediators in the endotoxin induced cascade, acute phase response and malignant cachexia. TGFβ is one of the few monocyte down-regulators; it inhibits some monocyte secretions and also other functions. IL-12, initially known as natural killer cell stimulatory factor demonstrates the important contribution made by products of the monocyte/macrophage population to the regulation of cellular and natural immunity[24,122,123]. Cytokine dysregulation has been reported in patients with sepsis, trauma, burns, inflammatory bowel diseases, severe acute pancreatitis, meningitis, alcoholic hepatitis and malaria. The cytokine levels have been reported to be related to the patients, prognosis[124–131]. The blocking of their production with anti-endotoxin antibodies or anti-monocyte-activators[132–134] or blocking of their actions with anti-cytokine antibodies or cytokine antagonists[70,135–136] has recently been reported to have a beneficial role in some of these diseases although these are at present controversial results[134].

Monocytes from jaundiced patients show an increased production of TNFα and IL-6, but not of IL-1 or TGFβ[31,137]. Two independent laboratories have reported the existence of soluble TNF antagonist/binding protein/inhibitor), a factor that binds to TNF and reduces its biological effects. Two forms of TNF soluble receptor have been reported (sTNFR-P55 and sTNFR-P75), these are natural TNF inhibitors that can bind to TNF and abolish its effects. Bemelmans *et al.*[138] reported from their animal study (mice) that after bile duct ligation, there are increased serum levels of both forms of soluble receptors, by ELISA assay, compared with controls. This is co-incident with increased levels of the biologically in-active form of TNF in the circulation, by ELISA. This confirmed the results from others[139] using a bioassay, that there are increased levels of TNF inhibitor in jaundiced humans. Jaundiced patients who have a poor immediate clinical outcome have an increased level of TNF in the circulation with increased production of TNF but relatively low levels of TNF inhibitor. The harmful effects of increased TNF production are diminished by binding to the inhibitor, when this balance is disturbed damage to the host could result. In patients with jaundice[139], there is an imbalance between the level of TNF inhibitor and the production of TNF by monocytes.

Blood levels of various cytokines have also been studied. Bemelmans 140 showed that in jaundiced animals, there are increased blood TNF and IL-6 levels which may suggest the production of these cytokines by the monocyte/macrophage is increased. In studies with jaundiced patients blood levels of IL-6, and TGFβ are also increased[142] and this may be related to the increased acute phase protein production in these patients as IL-6 is one of the most potent stimuli for acute phase protein synthesis by hepatocytes.

Platelet activation factor (PAF), an important mediator in inflammation and endotoxic shock, has recently been shown to be produced in large quantities by Kupffer cells and the expression of its mRNA increased in bile duct ligated rats[143]. This may be a result of portal endotoxaemia. The plasma level after common bile duct ligation in the rabbit, is also rapidly increased[144]. This peptide is not only a water and electrolyte regulator, but also an immune cell primer. In contrast to PAF and atrial natriueretic peptide, circulating levels of insulin-like growth factor I, (IGF-I) in jaundiced animal (rats in this instance) are decreased compared with controls[145].

From the literature it seems that, broadly speaking, in jaundiced patients immune function is inhibited, (almost certainly the RES, perhaps cellular and some aspects of neutrophil immunity). There are two factors however in these studies which raise some doubts. Firstly, there is some inconsistency in these studies and secondly, the conclusion that immune function is depressed in jaundiced patients is not in line with the fact that in these patients there is endotoxaemia, hyperbilirubinaemia, and other factors which are strong stimulators of immune cell function (see below). This area needs further clarification.

Apart from the systemic immune dysfunction, jaundiced patients also have local immunity dysfunction. Sung *et al.*[146] recently reviewed the biliary tract defence mechanisms against bacterial infection. These mechansims are: tight junctions between hepatocytes, Sphincter of Oddi, bile flow, bile salts in the gut, Kupffer cells, and secretory immunological responses particularly secretory IgA in the biliary and intestinal tract. Most of these mechanisms are damaged in jaundiced patients. Biliary obstruction raises the biliary pressure which causes damage to the tight junctions. Bile salt concentration increases in the circulation which can damage host immune function. Kupffer cell malfunction is discussed earlier as part of the RES. In addition, if biliary IgA cannot be secreted into the gut the increased level of circulating IgA may lead to increased deposition in the kidney causing renal damage.

### MEDIATORS RESPONSIBLE FOR THE IMMUNE FUNCTION CHANGES IN JAUNDICE

Almost 20 years ago Vierucci[104] showed that the depressed lymphocyte function in cholestatic patients could be reversed by normal human sera and cholestatic human sera could casue E rossettes with normal lymphocytes to be decreased. One or more factors in the serum will be responsible for the immune changes and although there are now more contenders than the bilirubin and bile salts suggested by these authors the full picture is still not yet clear.

### *Endotoxin*

There is increasing recognition of the existence of both portal and systemic endotoxaemia in obstructive jaundice. This has been confirmed both in animal studies [147,148,149] and in clinical studies[9,13,150–153]. Endotoxaemia has been postulated as one of the key factors in the pathophysiology of complications in jaundice, such as renal damage, coagulation disorders, and Multiple System Organ Failure (MSOF).

Possible contributors to endotoxaemia in these patients are as follows[149]:

(1) Absence of bile salts in the gut allows changes in the bacterial flora, and loss of the emulsifying anti-endotoxin effects of bile salts, which results in a large pool of endotoxin in the colon.

(2) Reduced Kupffer cell up-take of endotoxin which allows it to enter the systemic circulation, clearly shown by Clements *et al.*[40].

(3) Bacterial translocation can be promoted by factors, such as biliary infections, altered mucosal permeability and biliary obstruction[154].

Endotoxin initiates a cascade of pathophysiological events[71], involving most systems and organs. Apart from the well recognised pathways of endotoxin activating the complement system, the coagulation system, and a "direct" effect on blood elements[72], recent work has proved that endotoxin induces a cytokine cascade, including TNFα, IL-1, IL-6, and IL-8, these cytokines mediate further biological processes[73–76].

Greve[155] showed that endotoxin may contribute to macrophage activation in jaundice. In germ free rats, jaundice does not cause macrophage activation, but in normal rats there is activation of macrophages. Endotoxin has also been shown to stimulate β-glucuronidase production from macrophages and PAF production from Kupffer cells[143,156].

The role of endotoxaemia in TNF production in liver disorders has been further investigated by Badger *et al.*[157], who showed, in a rabbit model, that endotoxaemia in combination with impaired hepatic clearance of endotoxin may potentiate TNF production. This combination seems to operate in jaundice[31,60,137,140,142]. Although most work suggests that endotoxin stimulates monocyte/macrophage phagocytosis[77–81], others imply the opposite effects of LPS on different subpopulations of macrophages[82]. This area certainly deserves more indepth studies in jaundice.

### *Bile Salts*

The increase in serum bile salts in jaundiced patients has important pathological implications, as bile salts can have profound effects on immune parameters. Bile acids, chenodeoxycholate, deoxycholate, and ursodeoxycholate all have inhibitory effects on lymphocyte mitogenesis induced by various stimuli[158,159]. Lymphocyte proliferation and IL-2 production are also reversibly inhibited by these bile acids, but the cell surface receptor numbers are not affected[167]. Bile acids increase β-glucuronidase production *in vitro*[156]. This may partly explain the increased level in the blood and production from monocytes in patients with jaundice[48,56,60].

### *Bilirubin*

The *in vitro* and *in vivo* effects of a raised bilirubin are unfortunately rather controversial and less conclusive.

### *Inhibitory Effects*

*In vitro* bilirubin can neutralise migration inhibition in a tuberculin hypersensitivity study[160]. Neutrophil hexose-monophosphate shunt activity is an important prerequisite for microbiocidal function and bilirubin can inhibit this[164]. It can also inhibit the chemotactic activity of granulocytes[162]. Bilirubin can inhibit phytohaemoagglutinin stimulated proliferation of lymphocytes[162] *In vitro*. It can decrease the numbers of antibody forming cells[163] and inhibit antibody-dependent cellular cytoxicity[84]. There seems to be a relationship between serum bilirubin levels and cellular immune responses in jaundiced patients[85].

### *Stimulatory Effects*

Bilirubin can stimulate macrophages and granulocytes *in vitro* to phagocytose[165]. It can also increase Fc receptors on peritoneal macrophages[166]. The proportion of activated lymphocytes is increased after bilirubin infusion[161].

### *Other Factors*

Hanai[168] reported that thromboxane may be another mediator of importance in obstructive jaundice. Thromboxanes are also immune stimulators and monocyte/macrophages are major producer of these eicosanoids.

### SUMMARY OF IMMUNE FUNCTION IN JAUNDICE

Although significant achievements have been made, there remain a great many un-answered questions, controversies, and in some cases confusion. In the past immune suppression, depression, or inhibition were terms simply used to describe the immune functional state in these patients. This is clearly no longer appropriate as the situation is far more complex. In the last few years, the identification of a variety of immune regulators and other insights into immune systems (surface molecules and so on) has revealed that there exists an infinitely more complex immune regulation network, and therefore a single immune parameter, phagocytosis for example, is inadequate to describe or to reflect immune function. Many earlier papers describing RES depression have used an *in vivo* clearance of particles or pathogens. There is increasing criticism of this method of measuring immune function, as clearance of different particles may vary and reflect the complex biological response of the host including circulatory factors rather than just RES or immune functions[61].

The current literature suggests that in general, monocyte/macrophage secretory functions (cytokines, lysosomal enzymes, superoxide and prostaglandins) are up-regulated, while its phagocytic functions are either depressed or unchanged. Assays of cellular immunity may reveal depression. Neutrophil superoxide production is increased but changes in phagocytic function are not yet clear. A proposed mechanism may be that when biliary obstruction first appears, there is a transient or sustained endotoxaemia that may activate cytokine production from monocyte/macrophages and other immune cells. Endotoxin together with cytokines and other mediators may prime or activate immune functions and the inappropriately activated mechanisms may cause damage to the host. This mechanism is similar to that seen in sepsis and major trauma. The other biochemical changes, for examples bilirubinaemia and increased bile salts may induce other modifications of the immune system. The authors therefore recommend the term “immune malfunction” or “immune dysfunction” rather than immune suppression in these circumstances.

### MEANS TO RESTORE THE IMMUNE FUNCTION IN JAUNDICE

### *Biliary Drainage*

This may prove to be the best way to intervene, relieving both jaundice and restoring immune functions. Katz has shown that 2 weeks after choledochoduodenostomy in jaundiced animals, the impaired trapping of bacteria in the liver was restored and the increased trapping of bacteria in the lung was also reversed[32]. Surgical drainage (choledochoduodenostomy) may restore the decreased IGF-I levels in jaundiced animal[145]. Ding *et al.*[39] also showed that in jaundiced rats, 1 week after choledochoduodenostomy, the RES uptake of infused bacteria returned to normal. A certain interval of time is needed for the RES to recover. Thompson[107] also showed that depressed lymphocyte functions can be reversed either by internal or external drainage, but particularly internal drainage. Percutaneous transhepatic biliary drainage will reduce the secretory IgA levels and therefore improve the bile to serum ratio of IgA[116]. Internal biliary drainage may reverse the depressed lymphocyte functions[36, 37, 111].

There has been debate as to whether it is simply drainage of bile that is important or returning bile flow to the bowel[111,148,149].

Although biliary drainage seems best for restoring immune functions it takes time to show its effects, and it is important that intervention aimed directly at the immune system should also be considered, if possible.

### CHEMOINTERVENTION

### *Lactulose and Bile Salt*

Pinocytosis of colloidal carbon and phagocytosis of sheep red cells by Kupffer cells can be inhibited by cholic acid (CA) and chenodeoxycholic acid (CDCA) [156]. CA or CDCA plus endotoxin also inhibits the endocytic function. This suggests that bile salts as well as endotoxin influences Kupffer cell functions.

Lactulose[155] and bile salts (particularly deoxycholic acid)[169] may inhibit endotoxin induced TNF production by normal or jaundiced monocytes. CDCA and taurochenodeoxycholic acid also inhibit monocyte IL-1 and IL-6 production, whereas ursodeoxycholic acid and tauroursodeoxycholic acid are less effective[109,158]. This suggests that selective use of bile salts may be beneficial. The same group has also shown that both CDCA and UDCA particularly the former, have strong inhibitory effects on lymphocyte proliferation in the jaundiced animal. Indeed, lactulose and sodium deoxycholate have also been reported by Pain[170] to prevent the postoperative renal dysfunction in patients with obstructive jaundice.

Mannitol[171] has been shown to be beneficial in patients with obstructive jaundice. The exact role of CCK (cholecystokinin) in jaundice is not clear (although it is implicated that CCK may be related to the enlargement of eosinophilic granules)[172]. CCK however is greatly increased in jaundiced animal (rats) and CCK receptor antagonist have a beneficial effect in these jaundiced animals[173].

### *Anti-Endotoxin Treatment*

Early studies using Polymixin B to reduce endotoxin in the blood has proved to be inefficient in patients[174]. Shibayama[175] showed that anti-endotoxin agents, polymixin B, neomycin and lactulose failed to prevent endotoxin induced damage to the liver and biliary system.

Recently, Houldijk[176] reported that cholestyramine, an endotoxin binder may be used in preventing endotoxamia related renal damage in jaundice. In their animal studies (rats), they showed that by using cholestyramine, the reduced renal blood flow in jaundice may be significantly reversed. This may also prevent the further reduction of renal blood flow in jaundiced rats after surgery[177].

The use of anti-endotoxin antibody clinically may provide another approach. However, clinical trials using an antibody in patients with sepsis and other diseases has proved not to be of benefit to the patients.

### *RES Suppressants and Stimulants*

The precise status of the RES is not clear in jaundice and therefore both RES supressants and stimulants have been studied in these subjects.

Ding *et al.*[178] reported that by using a liposome encapsulated synthetic macrophage stimulus, muramyl dipeptide, it was possible to modify the impaired RES clearance of infused bacteria in jaundiced rats and also modify the depressed RES phagocytic index[178] by preventing bacterial translocation[179]. Pain[45] showed that a muramyl dipeptide analogue may improve the prolonged clearance of micro-aggregated human albumin in jaundice both in human and in rats.

Manipulating RES function with various depressants or stimulants has also been reported by Al-Tuwaijri[180]. The RES suppressant, methyl palmitate, modified liver damage by various agents, on the other hand the RES stimulant Glucan worsened the liver damage. Shibayama[181] has also shown that the RES suppressants, cortisone acetate, methyl palmitate and gadolinium chloride may also improve mortality and prevent endotoxin induced hepatotoxicity.

An extract of Tinospora Cordifolia has been shown to reverse the impaired neutrophil phagocytosis in jaundice (human and rat)[67]. Muramyl dipeptide also improves the impaired phagocytosis of mononuclear phagocytes[182].

Phagocytosis consists of a series of events, from opsonization, antigen recognition, antigen binding to cell surface, ingestion, acidification, fusion with lysosomes and subsequent killing and digestion. Although much research has shown the phagocytic defect in the RES, there is, however, no information about which part of the process is damaged and therefore the non-specific stimuli used so far may not have much beneficial effect. Indeed the reported work so far has failed to examine other immune functions after giving an immune stimulant. Such non-specific stimulants may, apart from enhancing the phagocytic process, also stimulate other unwanted effects such as toxic extracellular superoxide and lysosomal enzyme secretions leading to further damage to the tissues.

### *Other Agents*

Fumarulo[184] and Conese[185] showed that retinoids, anti-inflammatory agents, may inhibit the respiratory burst and degranulation in stimulated neutrophils and can also inhibit the procoagulant activity of mononuclear phagocytes, which has been shown to be increased in jaundice[46,83,86,87,88]. The clinical application of this for patients, however, needs to be further investigated.

Uchino showed that prophylactic infusion of activated protein C (APC) in jaundiced animals may significantly modify the hemorrhagic disorder that may be related to mononuclear and endothelial cell susceptibility to endotoxin[186,187].

Arginine, a T cells stimulator may modify the dysfunctioning lymphocytes in the immune system in jaundice as shown by Li[114]. Arginine has also been shown to modify delayed type hyper-sensitivity in jaundiced animals[183].

### *Cytokine Antibody and Antagonist*

The devastating effects of some cytokines (TNF, IL-1, IL-6 for example) have been shown in various diseases, sepsis, trauma, burns, inflammatory bowel diseases and rheumatoid arthritis. There are various studies using cytokine antibodies or antagonists to reduce these cytokine induced effects. Because the levels of various cytokines are raised in jaundiced patients and animals, anti-cytokine antibodies or cytokine antagonists may be a means to reduce cytokine mediated side effects.

Zhou[143] used PAF receptor antagonist in jaundiced animals and this reduced free radical levels and modified the liver damage seen in this model.

### *Essential Fatty Acid*

EFA's, as an immune regulator, have been used in a variety of clinical situations. Recent work[151,188] showed that one of the n-6 essential fatty acids, gamma linolenic acid and its lithium salt, have strong regulatory effects on both monocytes and neutrophils. The effects are dependent on the cell's functional status. EFA's inhibit TNF and hexosaminidase production from activated jaundiced monocytes, but stimulate TNF production from normal monocytes. Rheumatoid arthritis patients have a similar immune background to jaundiced patients, with activated monocyte/macrophages (proteolytic enzymes and cytokine production), neutrophils (reactive oxygen metabolite production), and lymphocytes (cytokine production). In recent years, both n-3 and n-6 fatty acids have been used in these patients and overall clinical and immunological responses are satisfactory. This approach may also be useful in jaundiced patients from the preliminary *in vitro* results.

### CONCLUSIONS

Patients with obstructive jaundice, have increased perioperative complications and significant immunological dysfunction or malfunction. While the phagocytic functions of the RES have been shown to be suppressed, other immune functions such as monocyte secretions, neutrophil superoxide productions, circulating cytokines are largely up-regulated. More work must be done to investigate further the details of these changes. Strategies should also be designed to restore normal immune function when this is appropriate or to allow the immune function to be optimised to deal with pathological situations such as sepsis.

### ABBREVIATIONS

| | |
|---|---|
| CCK | Cholecystokinin |
| CDCA | Chenodeoxycholic acid |
| GM-CSF | Granulocyte macrophage colony stimulating factor |
| IGF | Insulin-like growth factor |
| M-CSF | Macrophage colony stimulating factor |
| PHA | Phytoheamagglutinin |
| RES | Reticulo endothelial system |
| TNF | Tumour necrosis factor |
| CA | Cholic acid |
| EGF | Epidermal growth factor |
| IFN | Interferon |
| LPS | Lipopolysaccharide |
| PAF | Platelet activating factor |
| PMA | Phorbol myristate acetate |
| TGF | Transforming growth factor |
| TNFR | Tumour necrosis factor receptor |

### REFERENCES

1. Dixon, J.M., Armstrong, C.P., Duffy, S.W. and Davies, G.C. (1983). Factors affecting morbidity and mortality and after surgery for obstructive jaundice, a review of 373 patients. *Gut.*, **24**, 845–852.
2. Pain, J.A., Cahill C.J. and Bailey M.E. (1985). Perioperative complication in obstructive jaundice: therapeutic considerations. *Br J Surg.*, **72**, 942–945.
3. Grant, M.D, Jones R.C., Wilson S.E., Bombeck, C. T., Flint, L.M, Jonasson, O., Soroff H.S., Stellato T.A. and Dougherty, S.H. (1992). Single dose cephalosporin prophylaxis in high-risk patients undergoing surgical treatment of the biliary tract. *Surg Gynecol Obstetr.*, **174**, 347–354.
4. Holman, J.M., Rikkers L.F. and Moody, F.G. (1979). Sepsis in the management of complicated biliary disorders. *Am J Surg.*, **138**, 809–813.
5. Pitt H.A., Cameron, J.L., Positier, R.G. and Gadacz, T.R. (1981). Factors affecting mortality in biliary tract surgery. *Am J Surg.*, **141**, 66–72.
6. Armstrong, C.P., Dixon, J.M., Duffy, S.W. and Elton R.A., Davies, G. C. (1984). Wound healing in obstructive jaundice. *Br J Surg.*, **71**, 267–270.
7. Blamey, S.L., Fearson, K. C.H., Gilmour, W.H. and Osbourne, D.H., Carter, D.C. (1983). Prediction of risk in biliary surgery. *Br. J Surg.*, **70**, 535–538.
8. Greig, J.D., Krukowski, Z.H. and Matheson, N.A. (1988). Surgical morbidity and mortality in one hundred and twenty nine patients with obstructive jaundice. *Br J Surg.*, **75**, 216–219.
9. Hunt, D.R., Allison, M.E.M., Prentice, C.R.M. and Blumgart, L.H. (1982). Endotoxaemia, disturbance of coagulation and obstructive jaundice. *Am J Surg.*, **144**, 325–329.
10. Upyrev, A.V., Gusev, E.I.U., Il'ichev, V.A., Gorchakov, V.K. and Muratova, O.A. (1989). Acute peptic lesions of the gastroduodenal mucosa in patients with obstructive jaundice. *Khirurgiia*, **1**, 42–46.
11. Bailey, M.E. (1976). Endotoxin, bile salts and renal function in obstructive jaundice. *Br. J. Surg.*, **63**, 774–778.
12. Allison, M.E.M., Prentice, C.R.M., Kennedy, A.C. and Blumgart, L.H. (1979). Renal function and other factors in obstructive jaundice. *Br J Surg.*, **66**, 392–397.
13. Cahill, C.J. (1983). Prevention of postoperative renal failure in patients with obstructive jaundice the role of bile salts. *Br J Surg.*, **70**, 590–595.
14. Ozawa, K., Yamada, T., Tanaka, J. and Ukikusa, M., Tobe, T. (1979). The mechanism of suppression of renal function in patients and rabbits with jaundice. *Surg Gynecol Obstet.*, **149**, 54–60.
15. Keighley, M.R., Razay, G. and Fitzgerald, M.G. (1984). Influence of diabetes on mortality and morbidity following operations for obstructive jaundice. *Ann R Coll Surg Engl.*, **66**, 49–51.
16. Bayer, I. and Ellis, H. (1976). Jaundice and wound healing: an experimental study. *Br. J. Surg.*, **63**, 392–396.
17. Hunt, D.R. (1980). The identification of risk factors and their application to the management of obstructive jaundice. *Aust NZ Surg.*, **50**, 476–480.
18. Kunz, F., Amor, H., Hortnagl, G., Weiser, F. and Folzknecht, F., Braunsteiner, H. (1974). Disseminierte intravaskulare gerinnung und letale makrothombosierung bei einem patienten mit gallenwegskarzinom. *Dtsch Med Wschr.*, **99**, 2643–2647.
19. Johnston, R.B. (1989). Monocytes and macrophages. *New Engl J Med.*, **318**, 747–752.
20. Nathan, C.F. (1987). Secretory products of macrophages. *J Clin Invest.*, **79**, 319–326.
21. Nathan, C.F. (1990). Peptide growth factors and their receptors 2. (editors MB Sporn and AB Roberts), Springer, London, P427–462.
22. Malefyt, R.D.W., Abrams, J., Bennett, B., Figdor, C.G. and Vries J.E.D. (1991). Interleukin-10 (IL10) inhibits cytokine synthesis by human monocytes: an autoregulatory role of IL-10 produced by monocytes. *J Exp Med.*, **174**, 1209–1220.
23. Grotendorst, G.R., Smale, G. and Pency, D. (1989). Production of transforming growth factor beta by human peripheral blood monocytes and neutrophils. *J Cell Physiol.*, **140**, 396–402.
24. D'Andrea, A., Rengaraju, M., Valiante, N.M., Chehimi, J., Kubin, M., Aste, M., Chan, S.H., Kobayashi, M., Deborch, Y., Nickbarg, E., Chizzonite, R., Wolf, S.F. and Trinchieri, G. (1992). Production of natural killer cell stimulatory factor (interleukin-12) by peripheral blood mononuclear cells. *J Exp Med.*, 176, 1387–1398.
25. Sariban, E. and Kufe, D. (1988). Expression of the platelet-derived growth factor- and factor-II genes in human myeloid cell-lines and monocytes. *Cancer Res.*, **48**, 4498–4502.
26. Blanchard, D.K., Friedman, H., Klein, T.W. and Djeu, J.Y. (1989). Induction of interferon gamma and tumor necrosis factor by Legionella pneumophila. *J Leukocyte Biol.*, **45**, 538–545.
27. Halpern, B.N., Biozzi, G., Nicol, T. and Bilbert, D.L. (1957). Effect of experimental biliary obstruction on the phagocytic activity of the reticulo-endothelial system. *Nature*, **180**, 503–504.
28. Holman, J.M. and Rikkers, L.F. (1982). Biliary obstruction and host defense failure. *J. Surg Res.*, **32**, 208–213.
29. Ball, S.K., Grogan, J.B., Collier, B.J. and Scott-Conner, C. E. (1991). Bacterial phagocytosis in obstructive jaundice. A microbiologic and electron microscopic analysis. *Am Surg.*, **57**, 67–72.
30. Roughneen, P.T., Drath, D.B., Kulkarni, A.D. and Rowlands, B.J. (1987): Impaired nonspecific cellular immunity in experimental cholestasis. *Ann Surg.*, **206**, 578–582.
31. Adachi, Y., Arii, S., Sasaoki, T., Funaki, N., Higashitsuji, H., Fujita, S., Furutani, M., Mise, M., Zhang, W. and Tobe, T. (1992). Hepatic macrophage malfunction in rats with obstructive jaundice and its biological significance. *J. Hepatol.*, **16**, 171–176.
32. Katz, S., Yang, R., Rodefeld, M.J., Folkening, W.J. and Grosfeld, J.L. (1991). Impaired hepatic bacterial clearance is reversed by surgical relief of obstructive jaundice. *J. Pediatr Surg.*, **26**, 401–406.
33. Katz, S., Merkel G.J., Folkening W.J., Rosenthal R.S. and Grosfeld J.L. (1991). Impaired clearance and organ localization of *Cancida albicans* in obstructive jaundice. *J Pediatr Surg.*, **26**, 904–907.
34. Lee, E. (1972). The effect of obstructive jaundice on the migration of reticulo-endothelial cells and fibroblasts in to early experimental granulomata. *Br. J. Surg.*, **59**, 875–977.
35. Scott–Conner C.E., Grogan J.B., Scher K.S. and Bernstein J. (1989). Impaired clearance of Escherichia coli bacteremia in early biliary obstruction. *Am J Surg.*, **157**, 210–214.
36. Megison, S.M., Dunn, C.W, Horton, J.W. and Chao, H. (1989). Immunologic recovery after biliary drainage in obstructive jaundice. *Current Surg.*, **46**, 383–385.
37. Megison, S.M., Dunn, C.W., Horton, J.W. and Chao, H. (1991). Effects of relief of biliary obstruction on mononuclear phagocyte system function and cell mediated immunity. *Br J Surg.*, **78**, 568–571.
38. Tanaka, N., Ryden, S., Bergqvist, L., Christensen, P. and Bengmark, S. (1985): Reticulo-endothelial function in rats with obstructive jaundice. *Br J Surg.*, **72**, 946–949.
39. Ding, J.W., Anderson R., Stenram, U., Lunderquist, A. and Bengmark, S. (1992). Effects of biliary decompression on reticuloendothelial function in jaundiced rats. *Br J Surg.*, **79**, 648–662.
40. Clements, W.D., Halliday, M.I., Mc Caigue, M.D., Barclay, R.G. and Rowlands, B.J. (1993). Effects of extrahepatic obstructive jaundice on Kupffer cell clearance capacity. *Arch Surg.*, **128**, 200–205.
41. Allen, M.O., Wilton, P.B., Barke, R.A., Gerding, D.N., Forstrom, L.A., Shafer, R.B. and Vennes, A. (1989). Effects of biliary obstruction of hepatic clearance of bacteria. *Arch Surg*, **128**, 973–977.
42. Ohlsson, E.G. (1972). The effect of biliary obstruction on the distribution of the hepatic blood flow and reticuloendothelial system in dogs. *Acta Chir Scand*, **138**, 159–164.
43. Drivas, G., James, O. and Wardle, N. (1976). Study of reticuloendothelial phagocytic capacity in patients with cholestasis. *J. Br Med.*, **1**, 1568–1569.
44. Pain, J.A. (1987). Reticuloendothelial function in obstructive jaundice. *Br J Surg.*, **74**, 1091–1094.
45. Pain, J.A., Collier, D.S. and Ritson, A. (1987): Reticuloendothelial system phagocytic function in obstructive jaundice and its modification by a muramyl dipeptide analogue. *Eur Surg Res.*, **19**, 16–22.
46. Semeraro, N., Montemurro, P., Chetta, G., Altomare, D.F., Giordano, D. and Colucci, M. (1989). Increased procoagulant activity of peripheral blood monocyte in human and experimental obstructive jaundice. *Gastroenterol*, **96**, 892–989.
47. Hines, J.E., Sohnson, S.J. and Burt, A.D. (1993). In vivo response of macrophages and perisinusoidal cells to cholestatic liver injury. *Am J Path.*, **142**, 511–518.
48. Hultberg, B., Braconier, J.H., Isaksson, A., and Jansson, B. (1981). ß-hexosaminidase level in serum from patients with viral heptatitis as a measure of reticulo endothelial function. *Scan J Infect Dis.*, **13**, 241–245.
49. Okado, S. and O'Brein, J.S. (1969). Tay-Sachs disease: generalised absence of a beta-D-N-acetylhexosaminidase component. *Science*, **165**, 698–700.
50. Sandoff, D. (1969). Variation of ß-N-acetylhexosaminidase-pattern in Tay-sachs disease. *FEBS Lett.*, **4**, 351–354.
51. Tonnesen, T., Andersen, P. and Guttler, F. (1988). Highly increased levels of serum ß-hexosaminidase, arylsulphatase A and ß-galactosidase in a patient with sepsis. *J Inher Metab Dis.*, **11**, 428–429.
52. Shattuck, K.E., Richardson, C.J., Rassin, D.K. and Lobe, T.E. (1987). Evaluation of hexosaminidase activity as a potential biochemical marker in serum for necrotizing enterocolities. *J Pediatr Gastroenterol Nutr.*, **6**, 234–237.
53. Tsao, D., Freeman, H.J. and Kim, Y.S. (1979). β-hexosaminidase isoenzymes in tissues, cultured cells and media from human fetal intestine and colonic adenocarcinoma. *Cancer Res.*, **39**, 3405–3410.
54. Alhadeff, J.A. and Holzinger, R.T. (1982). Presence of an atypical thermolabile species of β-hexosaminidase B in metastatic-tumour tissue of human liver. *J. Biochem.*, **201**, 95–99.
55. Brattain, M.G., Kimball, P.M., Durant, J.R., Pretlow, II T.G., Smith, D., Carpentar, J. and Marks, M. (1979). Urinary hexosamindase in patients with lung carcinoma. *Cancer*, **44**, 2267–2272.
56. Scapa, E., Novis, B.H., Loewenstein, M., Thomas, P. and Zamcheck, N. (1988). Serum β-N-acetyl hexosaminidase and bile acid levels in patients with benign and malignant biliary obstruction. *Dig Dis Sci.*, **33**, 189–192.
57. Jessup, W. and Dean, R.T. (1980). Spontaneous lysosomal enzyme secretion by a murine macrophage like cell line. *J. Biochem.*, **190**, 847–850.
58. Bourbouze, R., Raffi, F., Dameron, G., Hali-Miraftab, H., Loko, F. and Vilde, J.L. (1991). N-acetyl-ß-D-glucosaminidase (NAG) isoenzymes release from human monocyte-derived macrophages in response to zymozan and human recombinant interferon-a. *Clin Chim Acta.*, **199**, 185–194.
59. Leoni, P. and Dean, R.T. (1983). Mechanisms of lysosomal enzyme secretion by human monocytes. *Biochim Biophys Acta.*, 762, 378–389.
60. Jiang, W.G. and Puntis, M.C.A. (1993). Monocyte secretion of hexosaminidase in patients with obstructive jaundice. *HPB Surg.*, **7**, 15–23.
61. Frank, M.M. (1990). The role of macrophages in blood stream clearance. In 'Human monocytes', Edited by Zembala M and Asheron GL. Academic Press, London, Pages 337–344.
62. Guthrie, L.A., McPhail, L.C., Henson, P.M. and Johnson, R.B. (1984). Priming of neutrophils for enhanced release of oxygen metabolites by bacterial lipopolysaccharide. *J Exp Med.*, **160**, 1656–1671.
63. Berkow, R.L., Wang, D., Larrick, J.W., Dodson, R.W. and Howard, T.H. (1987). Enhancement of neutrophil superoxide production by preincubation with recombinant human tumor necrosis factor. *J Immunol*, **139**, 3783–3791.
64. Cross, A.S., Lowell, G.H., Palmblad, J., Sadoff, S.C., Young, L. and Berger, M. (1985). Mechanisms of priming of human neutrophils by a soluble lymphoblastoid cell factor. *J Immunol*, **139**, 3783–3791.
65. Kelbanoff, S.J., Vadas, M.A., Harlan, J.M., Sparks, L.H., Gamble, J.R., Agosti, J.M. and Waltersdorph, A.M. (1986). Stimulation of neutrophils by tumor necrosis factor. *J Immunol*, **136**, 4220–4225.
66. Roughneen, P.T.M., Drath, D.B., Kulkarni, A.D., Kuma, S.C., Andrassy, R.J. and Rowlands, B.J. (1989). Inflammatory cell function in young rodents with experimental cholestasis: investigation of functional deficits, their aetiology and their reversibility. *J Pediatr Surg.*, **24**, 668–673.
67. Rege, N.N., Nazqreth, H.M., Bapat, R.D. and Dahanukar, S.A. (1989). Modulation of immunosuppression in obstructive jaundice by Tinospora Cordifolia. *Ind J Med Res.*, **90**, 478–483.
68. Andy, OJ Jr, Grogan, J.B., Griswold, J.A., Scott-and Conner, C.E. (1992). Peritoneal neutrophil chemotaxis is impaired in biliary obstruction. *Am Surg.*, **58**, 28–31.
69. Scapa, E., Thomas, P. Lowenstein, M.S. and Zamcheck,

N. (1985). Serum β-N-acetyl hexosaminidase (β-NAH) as a discriminant between malignant and benign extrahepatic biliary obstruction: comparison with carcinoembryonic antigen (CEA). *Eu J Cancer Clin Oncol.*, **21**, 1037–1042.

70. Starnes, H.F., Pearce, M.K., Tewari, A., Yim, J.H., Zou, J.C. and Abams, J.S. (1990). Anti-IL-6 monoclonal antibodies protect against lethal Escherichia Coli infection and lethal tumor necrosis factor-alpha challenge in mice. *J Immunol*, **149**, 4185–4191.
71. Young, L.S. (1991). Exdotoxins and mediators. in "Bacterial endotoxins. Cytokine mediators and new therapies for sepsis", Editors Sturk A, van Deventer S. J. H. *et al.* 1992, by Wiley-Liss. New York. Pages 1–7.
72. Young, L.S. (1989). Gram-negative sepsis. In "Principles and practice of infectious dieases" 3rd edition. by Mandell G. John Wiley and Sons, New York. Pages 611–635.
73. Michie, H.R., Manogue, K.R. and Spriggs, D.R. *et al.* (1988). Detection of circulating tumor necrosis factor after endotoxin administration. *N Eng J Med.*, **318**, 1481–1486.
74. Dinarello, C.A. (1989). Interleukin-1 and its biologically related cytokines. *Adv immunol*, **44**, 153.
75. Schindler, R., Mancilla, J., Endress, S., Ghorbani, R., Clark, S.C. and Dinarello, C.A. (1990). Correlations and interactions in the production of interleukin-6 (IL-6), IL-1, and tumor necrosis factor (TNF) in human blood mononuclear cell. *Blood*, **75**, 40–47.
76. van Deventer, S.J.H. and Cerami, A. (1992). Novel lipopolysaccharide-induced cytokine. in "Bacterial endotoxic lipopolysaccharides". Editors Ryan, J.L. and Morrison, D.C. by CRC Press, Boca Raton. Pages 197–206.
77. Cardozo, C., Edelman, J. and Lesser, M. (1992). Lipopolysaccharide-induced stimulation of alveolar macrophage opsonin-independent phagocytosis. *J Surg Res.*, **53**, 170–174.
78. Higginbotham, J.N., Lin, T.L. and Pruett, S.B. (1992). Effect of macrophage activation on killing of listeria monocytogen. *Clin Exp Immunol*, **88**, 492–498.
79. Belosevic, M. and Daniels, C.W. (1992). Phagocytosis of Giardia Lamblia trophozoites by cytokine-activated macrophages. *Clin Exp Med.*, **87**, 304–309.
80. Griffin, F.M.Jr. and Mullinax, P.J. (1990). High concentrations of bacterial lipopolysaccharide, but not microbial infection-induced inflammation, activate macrophage C3 receptors for phagocytosis. *J Immunol*, **145**, 69–701.
81. Sanstrom, T., Bjermer, L. and Rylander, R. (1992). Lipopolysachharide (LPS) inhalation in healthy subjects increases neutrophils, lymphocytes and fibronectin levels in bronchoalveolar lavage fluid. *J. Eur Resp.*, 5, 992–996.
82. Ohki, K., Soejima, T., Kohashi, O. and Nagayama, A. (1991). Opposite effects of bacterial lipopolysaccharide on Fc-receptor-mediated phagocytosis of two bone marrow-derived macrophage cell lines, BDM-1 and BDM-1 W3. *Cell Struc Funct.*, **16**, 495–502.
83. Levy, R., Schlaeffer, F., Keynan, A., Nagauker, O., Yaari, A. and Sikuler. (1993). Increased neutrophil function induced by bile duct ligation in a rat model. *Hepatol.*, **17**, 908–914.
84. Meisel, P. and Jahr, H. (1991). Influence of albumin-bound bilirubin on the antibody-dependent cellular cytoxicity of human cells in vitro. *Biol Neonate.*, **60**, 308–313.
85. Cainzos, M., Alcalde, J.A., Potel, J. and Puente, J.L. (1990). Variation in the cellular immune response as a function of total bilirubin. *Surg Res Coomun.*, **7**, 285–299.
86. Roughneen, P.T., Drath, D.B., Kulkarni, A.D. and Rowlands, B.J. (1987): Impaired nonspecific cellular immunity in experimental cholestasis. *Ann Surg.*, **206**, 578–582.
87. Jiang, W.G., Hallett, M.B. and Puntis, M.C.A. (1994). Neutrophil priming by cytokines in patients with obstructive jaundice. *HPB Surg.*, **7**, 281–289.
88. Puntis, M.C.A., Jiang, W.G. and Hallett, M.B. (1992). Neutrophil oxidative response and its relationship to clinical outcomes in jaundiced patients. *HPB Surg.*, **5** (Suppl), 204.
89. Dahm, L.J. and Roth, R.A. (1990). Differential effects of lithocholate on rat neutrophil activation. *J Leukocyte Biol.*, **47**, 551–560.
90. Schmeling, D.J., Oldham, K.T., Guice, K.S., Kunkel, R.G. and Johnson, K.J. (1991). Experimental obliterative cholangitis. A model for the study of biliary atresia. *Ann Surg.*, **213**, 350–355.
91. Swain, M.G., Tjandra, K., Kanwar, S. and Kubes, P. (1995). Neutrophil adhesion is impaired in a rat model of cholestasis. *Gastroenterol*, **109**, 923–932.
92. Yuo, A., Kitagawa, S., Motoyoshi, K., Asuma, E., Saito, M. and Takaku, F. (1992). Rapid priming of human monocytes by human hemotopoietic growth factors-granulocyte-macrophage colony-stimulating factor (CSF), macrophage-CSF, and interleukin-3 selectively enhance superoxide release triggered by receptor-mediated agonists. *Blood*, **79**, 15433–1557.
93. Doerfler, M.E., Danner, R.L., Shelhamer, J.H. and Parrillo, J.E. (1989). Bacterial lipopolsaccharide primes human neutrophils for enhanced production of leukotriene B4. *J Clin Invest.*, **83**, 970–977.
94. Davis, J.M., Meger, J.D., Barie, P.S., Yurt, R.W., Duhaney, R., Deneen, P. and Shires, G.P. (1990). Elevated production of neutrophil leukotriene B4 precedes pulmonary failure in critically ill surgical paitents. *Surg Gynecol Obstet.*, **170**, 495–500.
95. Fantone, J.C. and Ward, P.A. (1985). Polymorphonuclearleukocyte-mediated cell and tissue injury: oxygen metabolites and their relations to human disease. *Hum Pathol.*, **16**, 973–978.
96. Tanaka, H., Ogura, H., Yokota, J., Sugimoto, H., Yoshioka, T. and Sugimoto T. (1991). Acceleration of superoxide production from leukocytes in trauma patients. *Ann Surg.*, **214**, 187–192.
97. Tanaka, H., Sugimoto, H., Yoshioka, T. and Sugimoto, T. (1991). Role of granulocyte elastase in tissue injury in patients with septic shock complicated by multiple-organ failure. *Ann Surg.*, **213**, 81–85.
98. Dham, L.J., Schultze, A.E. and Roth R.A. (1991). Activated neutrophils injure the isolated rat liver by an oxygen radical-dependent mechanism. *Am J Pathol.*, **139**, 1009–1020.
99. Kowanko, I.C. and Ferrante, A. (1991). Granulocyte-macrophage colony-stimulating factor augmentsneutrophil-mediated cartilage degradation and neutrophil adherence. *Arthritis Rheumatism*, **34**, 1452–1460.
100. Mundi, H., Bjorksten, B., Svanborg, C., Ohman, L. and Dahlgren, C. (1991). Extracellular release of reactive oxygen species from human neutrophils upon interaction with Escherichia coli strains causing renal scarring. *Infect Immun.*, **59**, 4168–4172.
101. Trautinger, F., Hammerle, A.F., Poschl, G. and Michsche, M. (1991). Respiratory burst capability of polymorphonuclear neutrophils and TNF-α serum levelsin relationship to the development of septic syndrome in critically ill patients. *J Leukocyte Biol.*, **49**, 449–454.
102. Gross, V., Scholmerich, J., Lesser, H.G., Salm, R., Lausen M., Ruckarer, K., Schoffel, U., Lay, L., Heinisch, A., Farthman and Gerok, W. (1990). Granulocyte elastase in assessment of severity of acute pancreatitis. *Dig Dis Sci.*, 97–105.
103. Gross, V., Anderseen, R., Leser, H.G., Ceska, M., Liehl, E., Lausen, M., Fartmann, E.H., and Scholmerich J. (1992).

Interleukin-8 and neutrophil activity in acute pancreatitis. *Eu J Clin Invest.*, **22**, 200–203.

104. Vierucci, A., De Martino, M., Novembre, E., Surrenti, C., Gabrielli, M. and Biadaioli, R (1977). Effect of the serum of patients with cholestasis on lymphocyte E rosette formation. *Ann Scalvo*, **19**, 1119–1129.

105. Greve, J.W., Gouma, D.J., Soeters, P.B. and Buurman, W.A. (1990). Suppression of cellular immunity in obstructive jaundiceis caused by endotoxin: a study with germ free rats. *Gastoenterol*, **98**, 478–485.

106. Vane, D.W., Redlich, P., Weber, T., Leapman, S., Siddiqui, A.R. and Grossfeld, J.L. (1988). Impaired immune function in obstructive jaundice. *J Surg Res.*, **45**, 287–293.

107. Thompson, R.L., Hoper, M., Diamond, T. and Rowlands, B.J. (1990). Development and reversibility of Tlymphocyte dysfunction in experimental obstructive jaundice. *Br J Surg.*, **77**, 1229–1232.

108. Fraser, I.A., Krakowka, S., Ringler, S., Carey, L.C. and Ellison, E.C. (1989). Lymphocyte function in obstructive jaundice. *Am J Surg.*, **157**, 405–409.

109. Calmus, Y., Weill, B., Ozier, Y., Chereau, C., Houssin, D. and Poupon, R. (1992). Immunosuppressive properties of chenodeoxycholic and ursodeoxycholic acids in the mouse. *Gastroenterol*, **103**, 617–621.

110. Roughneen, P.T., Kumar, S.C., Pellis, N.R. and Rowlands, B.J. (1988). Endotoxaemia and Cholestasis. *Surg Gynecol Obstetr.*, **167**, 205–210.

111. Roughneen, P.T., Gouma, D.J., Kulkarni, A.D., Fanslow, W. F. and Rowlands, B. J. (1986): Impaired specific cell-mediated immunity in experimental biliary obstruction and its reversibility by internal biliary drainage. *J Surg Res.*, **41**, 113–125.

112. Feduccia, T.D., Scott-Conner, C.E. and Grogan, J.B. (1988): Profound suppression of lymphocyte function in early biliary obstruction. *Am J Med Sci.*, **296**, 39–44.

113. Li, H., Xiong, S.T., Zhang, S.X., Liu, S.B., Zou, P.N. and Xiang, J.P. (1991): Altered lymphocyte subsets and natural killer cells of patients with obstructive jaundice in perioperative period. *J Tongji Med Univ.*, **11**, 145–149.

114. Li, H., Xiong, S.T., Zhang, S.X., Luo, Y. and Feng, Z.H. (1991): Effect of arginine on immune function in rats with obstructive jaundice. *J Tongji Med Univ*, **11**, 150–154.

115. Wagner, F., Assemi, C., Lersch, C., Hart, R. and Classen, M. (1990). Soluble interleukin-2 recepetor and soluble CD8 in liver cirrhosis and obstructive jaundice. *Clin Exp Immunol*, **82**, 344–349.

116. Ohshio, G., Furukawa, F., Manabe, T., Tobe, T. and Hamashima, Y. (1986): Relationship between secretory IgA, IgA-containing (C3-fixing) circulating immune complexes, and complement components) (C3, C4) in patients with obstructive jaundice. *Scand J Gastroneterol*, **21**, 151–157.

117. Ohshio, G., Manable, T., Tobe, T., Yoshioka, H. and Hamashima, Y. (1988): Circulating immune complex, endotoxin, and biliary infection in patients with biliary obstruction. *Am J Surg.*, **155**, 343–347.

118. Kawaguchi, K. and Koike, M. (1987): Glomerular alterations associated with obstructrive jaundice. *Hu Pathol.*, **18**, 1149–1154.

119. Nakanuma, Y. and Yoshida, K. (1988): Expression of beta 2-microglobulin on interlobular bile ducts in primary biliary cirrhosis and other hepatobiliary diseases. *Acta Pathol Jap.*, **38**, 853–860.

120. Emanicpator, S.N., Gallo, G.R., Raxaboni, R. and Lamm, M.E. (1983): Experimental cholestasis promotes the deposition of glomerular IgA immune complexes. *Am J Pathol.*, **113**, 19–26.

121. Benamouzig, R., Buffet, C., Fourre, C., Ink, O., Moati, F. and Etienne, J.P. (1989). Evaluation of the serum level of antigen CA-19.9 during the course of extra-heptaic cholestasis. *Gastroenterol Clin Biol.*, **13**, 2BIS.

122. Gately, M.K., Desai, B.B., Wolitzky, A.G., Quinn, P.M., Dwyer, C.M., Podlaski, F.J., Familletti, P.C., Sinigaglia, F., Chizonnite, R, Gubler, U. and Stern, A.S. (1991). Regulation of human lymphocyte proliferation by a heterodimeric cytokine, IL-12 (cytotoxic lymphocyte maturation factor). *J Immunol*, **14**, 874–882.

123. Bertagnlli, M.M., Lin, B.Y., Young, D. and Herrman, S.H. (1992). IL-12 augments antigen dependent proflieration of activated T lymphocytes. *J Immunol*, **149**, 3778–3783.

124. Damas, P., Reuter, A., Gysen, P., Demonty, J., Lamy, M. and Franchimont, P. (1989). Tumor necrosis factor and interleukin-1 serum levels during severe levels during severe sepsis in humans. *Crit Care Med.*, **149**, 3778–3783.

125. Mozes, T., Ben-Effraim, S., Tak, C.J.A. M., Heiligers, J.P.C., Saxena, P.R. and Bonta, I.L. (1991). Serum levels of tumor necrosis factor determine fatal or non-fatal course of endotoxic shock. *Immunol Lett.*, **27**, 157–162.

126. Takayama, T.K., Miller, C. and Szabo, G. (1990). Elevated tumor necrosis factor α production concomitant to elevated prostaglandin $E_2$ production by trauma patients monocytes. *Arch Surg.*, **125**, 29–35.

127. Nijsten, M.W.N., Hack, C.E., Helle, M., Ten Duis, H.J., Klasen, H.J. and Aarden, L.A. (1992). Interleukin-6 and its relation to the humoral immune response and clinical parameters in burnt paitents. *Surg.*, **109**, 761–767.

128. Mazlam, M.Z. and Hodgson. (1992). Peripheral blood monocyte cytokine production and acute phase response in inflammatory bowel disease. *Gut.*, **33**, 773–778.

129. Exley, A.R., Leese, T., Holliday, M.P., Swann, R.A. and Cohen, J. (1992). Endotoxaemia and serum tumour necrosis factor as prognostic markers in severe acute pancreatitis. *Gut.*, **33**, 1126–1128.

130. Sheron, N., Lau, J., Daniels, H., Alexander, G.J. and Williams, R. (1991). Increased plasma tumor necrosis factor alpha in chronic hepatitis B virus infection. *Hepatol*, 12, 241–245.

131. Sheron, S., Bird, G., Goka, J., Alexander, G. and Williams, R. (1991). Elevated plasma interleukin-6 and increased severity and mortality in alcoholic hepatitis. *Clin Exp Immunol*, **84**, 449–453.

132. O'Riodain, M.G., Collins, K.H., Pilz, M., Saporoschetz, I.B., Mannick, J.A. and Rodrick, M.L. (1992). Modulation of macrophage-hyperactivity improves survival in a burn-sepsis model. *Arch Surg.*, **127**, 152–158.

133. Redmond, H.P., Chavin, K.D., Bromberg, J.S. and Daly, J.M. (1991). Inhibition of macrophage activating cytokines is beneficial in the acute septic response. *Ann Surg.*, **214**, 502–509.

134. Inglis, T.J.J., Hawkey, P.M., Lacey, R.W., Bodenham, A., Kay, E. and Calvert, R.T. (1993). Monoclonal antiendotoxin agent HA-1A (controxin). *Lancet.*, **341**, 303.

135. Walsh, C.J., Sugerman, H.J., Mullen, P.G., Carey, P.D., Leeper-Woodford, S.K., Jesmok, G.J. Ellis, E.F. and Fowler, A.A. (1992). Monoclonal antibody to tumor necrosis factor a attenuates cardiopulmonary dysfunction in porcine gram-negative sepsis. *Arch Surg.*, **127**, 138–145.

136. Beutler, B., Milsark, I.W. and Cerami, A. (1985). Passive immunization against cachectin/tumor necrosis factor protects mice from lethal effect of endotoxin. *Science*, **229**, 868.

137. Puntis, M.C.A. and Jiang, W.G. (1992). Monocytes from obstructive jaundice patients show increased TNF and IL-6 production. *Br J Surg.*, **79**, 458–459.

138. Bemelman, M.H.A., Buurman, W.A. and Gouma, D.J. (1993). The presence of TNF and soluble TNF receptors in obstructive jaundice. *Gastroenterol*, **104**, suppl, A665.

139. Puntis, M.C.A. and Jiang, W.G. (1993). Imbalance between TNFα and TNF inhibitor in patients with obstructive jaundice. *HPB Surg.*, **6**, Suppl, 87.

140. Bemelmans, M.H.A., Gouma, D.J., Greve, J.W. and

Buurman, W.A. (1992). Cytokines tumor-necrosis factor and interleukin-6 in experimental biliary obstruction in mice. *Hepatol.*, **15**, 1132–1136.

141. Puntis, M.C.A. and Jiang, W.G. (1996). Blood cytokine level and monocyte activation in paitents with obstructive jaundice. *J Gastroenterol Hepatol.*, **11**, 7–13.

142. Kimura, F., Miyazaki, M., Suwa, T., Hayashida, K. and Kakizaki, S. (1992). Hepatic protein synthesis and cytokines in obstructive jaudice. *HPB Surg.*, **5**, Suppl. 126.

143. Zhou, W., Chao, W., Levine, B.A. and Olson, M.S. (1992). Role of platelet-activating factor in hepatic responses after bile duct ligation in rats. *Am J Physiol.*, **263**, 587–592.

144. Valverde, J., Martinez-Rodenas, F., Pereira, J.A., Carulla, X., Jimenez, W., Gubern, J.M. and Sitges-Serra, A. (1992). Rapid increase in plasma levels of atrial natriuretic peptide after common bile duct ligation in the rabbit. *Ann Surg.*, **128**, 200–205.

145. Katz, S., Pescobitz, O.H. and Grosfeld, J.L. (1991). Growth failure and decreased levels of insulin-like growth factor I in obstructive jaundice are reversed by bile diversion. *J Pediatr Surg.*, **26**, 900–903.

146. Sung, J.Y., Costerton, J.W. and Shaffer, E.A. (1992). Defense system in the biliary tract against bacterial infection. *Dig Dis Sci.*, **37**, 689–696.

147. Gouma, D.J., Coelho, J.C.U., Fisher, J.D., Schlegel, J.F., Li, Y. F. and Moody, F.G. (1986). Endotoxaemia after relief of biliary obstruction by internal and external drainage in rats. *Am J Surg.*, **151**, 476–479.

148. Diamond, T., Dolan, S., Thompson, R.L.E. and Rowlands, B.J. (1990). Development and reversal of endotoxaemia and endotoxin-related death in obstructive jaundice. *Surg.*, **108**, 370–375.

149. Diamond, T. and Rowlands, B.J. (1991). Endotoxaemia in obstructive jaundice. *HPB Surg.*, **4**, 81–94.

150. Wardle, E.N. and Wright, N.A. (1970). Endotoxin and acute renal failure associated with obstructive jaundice. *J. Br Med.*, **4**, 472–474.

151. Jiang, W.G, Hallet, M.B., Scott, C., Horrobin, D.F. and Puntis, M.C.A. (1996). Inhibition of neutrophil respiratory burst and cytokine priming by gamma linolenic acid. *Br J Surg.*, **83**, 659–667.

152. Pain, J.A. and Bailey, M.E. (1986). Experimental and clinical study of lactulose in obstructive jaundice. *Br J Surg.*, **73**, 775–778.

153. Gawley, W.F., Gorey, T.F., Johnson, A.H., Collins, P.B., Osborne, D.H., Lane, B.E. and Collins, P.G. (1988). The effect of oral bile salts on serum endotoxin and renal functions in obstructive jaundice. *Br J Surg.*, **75**, 600.

154. Ding, J.W., Andersson, R., Soltesz, V. and Benmark, S. (1992). Bacterial translocation in experimental biliary obstruction. *HPB Surg.*, **5**, Suppl, 5.

155. Greve, J.W., Gouma, D.J., von Leeuwen, P.A. and Buurman, W.A. (1990). Lactulose inhibits endotoxin induced tumour necrosis factor production by monocyte. An in vitro study. *Gut.*, **31**, 198–203.

156. Takiguchi, S. and Koga, A. (1988). Effects of bile acids and endotoxin on the function and morphology of cultured hamster Kupffer cells. *Virchow Arch (Cell Biol)*, **54**, 303–311.

157. Badger, I.L.R., Townsend, P. and Buckels, J.A. (1992). Tumour necrosis factor alpha secretion in leporine endotoxaemia: role of the liver and effects of hepatic ischemia. *Gut.*, **33**, 694–697.

158. Calmus, Y., Gueochot, J., Podevin, P., Bonnefis, M.T. and Giboudeau, J. (1992). Differential effects of chenodeoxycholic and ursodeoxycholic acids on interleukin-1, interleukin-6 and tumor necrosis factor alpha production by monocytes. *Hepatol.*, **16**, 719–723.

159. Keane, R.M., Gadacz, T.R., Munster, A.M., Birmingham, W. and Winchurch, R.A. (1984): Impairment of human lymphocyte function by bile salts. *Surg.*, **95**, 439–443.

160. Svejcar, J., Miler, I. and Pekarek, J. (1984): Effect of bilirubin on an in vitro correlate of cell-mediated immunity– the migration inhibition test. *J Clin Lab Immunol*, **13**, 145–149.

161. Miler, I., Sima, P., Vetvicka, V., Indrova, M. and Slavikova, M. (1988). The potential immunosuppressive effect of bilirubin. *Allerg Immunol*, **34**, 177–184.

162. Rola-Plezcynski, M., Hensen, S.A., Vincent, M.M. and Bellanti, J.A. (1975): Inhibitory effects of bilirubin on cellular immune responses in man. *J Pediatr.*, **86**, 690–696.

163. Sima, P., Mala, J., Miler, I., Hodr, R. and Truxova, E. (1980): The suppressive effect of continuous infusion of bilirubin on the immune response in mice. *Folia Microbiol.*, **25**, 483–490.

164. Thong, Y.H. and Rencis, V. (1977): Bilirubin inhibits hexose-monophosphate shunt activity of phagocytosing neutrophils. *Acta Paediatr Scand*, **66**, 757–759.

165. Bilej, M., Vetvicka, V. and Sima, P. (1989). The stimulatory effect of bilirubin on phagocytotic activity. *Folia Microbiol.*, **34**, 136–140.

166. Vetvicka, V., Fornusek, L., Sima, P., Bilej, M., Taborsky, L., Rihova, B., Simeckova, J. and Miler, I. (1988). Effects of bilirubin on murine peritoneal and spleen cells. *Acta Pathol Microbiol Immunol Scand*, **96**, 671–675.

167. Lacaille, F. and Paradis, K. (1993). The immunosuppressive effect of ursodeoxycholic acid: a comparative *in vitro* study on human peripheral blood mononuclear cells. *Hepatol*, **18**, 165–172.

168. Hanai, T., Yura, J., Ogino, K., Hori, K. and Suzuki, T. (1989): Thromboxane as a possible hepatotoxic factor increased by endotoxemia in obstructive jaundice. *Jap J Surg.*, **19**, 556–562.

169. Greve, J.W., Dirk, D.J. and Buurman, W. (1989). Bile acids inhibits endotoxin-induced release of tumor necrosis factor by monocytes: an *in vitro* study. *Hepatol*, **10**, 454–458.

170. Pain, J.A., Cahill, C.J., Gilbert, J.M., Johnson, C.D., Trapnell, J.E. and Bailey, M.E. (1991). Prevention of postoperative renal dysfunction in patients with obstructive jaundice: a multicentre study of bile salts and lactulose. *Br J Surg.*, **78**, 467–469.

171. Gubern, J.M., Martinez-Rodenas, F. and Sitges-Serra, A. (1990): Use of mannitol as a measure to prevent postoperative renal failure in patients with obstructive jaundice. *Am J Surg.*, **159**, 144–445.

172. Tangoku, A., Doi, R., Chowdhury, P., Blevins, G.T., Jr., Pasley, J.N., Chang, L.W. and Rayford, P.L. (1993): Humoral factors that induce alterations of the pancreas in rats with obstructive jaundice. *Pancreas*, **8**, 103–108.

173. Tangoku, A., Doi, R., Chowdhury, P., Pasley, J.N., McKay, D.W. and Rayford, P.L. (1992): Use of a specific cholecystokinin receptor antagonist (L-364, 718) to determine the role of cholecystokinin on feeding and body weight in rats with obstructive jaundice. *J Ass Acadc Minor Physician*, **3**, 38–40.

174. Ingoldby, C.J.H. (1980). The value of polymyxin B in endotoxaemia due to obstructive jaundice and mesenteric ischaemia. *Br J Surg.*, **67**, 565–567.

175. Shibayama, Y (1989): Endotoxaemia and hepatic injury in obstructive jaundice. *J Pathol*, **159**, 335–339.

176. Houdijk, A., van Leeuwen, P., Boermeester, M. and Wesdorp, R. (1993). Reduced splanic blood flow following surgery in obstructive jaundice is prevented by enteral cholestyramine. *Gastroenterol*, **104**, Suppl. A254.

177. Houldijk, A., van Leeuwen, P., Boermeester, M. and Wesdorp, R. (1993). Enteral cholestyramine prevent fall in renal blood flow following surgery in obstructive jaundice. *Gastroenterol*, **104**, Suppl, A365.

178. Andersson, R., Ding, J.W., Hultberg, B. and Bengmark, S. (1992). Modification of reticuloendothelial function by muramyl dipeptide encapsulated liposomes in jaundiced rats treated with biliary decompression. *HPB Surg.*, **5**, Suppl, 15.

179. Ding, J.W., Andersson, R., Hultberg, B., Soltesz, V. and Bengmark, S. (1993). Modification of reticuloendothelial function by muraryl dipeptide-encapsulated liposomes in jaundiced rats treated with biliary decompression. *Scand J Gastroenterol*, **28**, 53–62.

180. Al-Tuwaijri, A., Akdamar, K. and Di Luzio, N.R. (1981): Modification of galactosamine-induced liver injury in rats by reticuloendothelial system stimulation or depression. *Hepatol*, **1**, 107–113.

181. Shibayama, Y., Hashimoto, K. and Nakata, K. (1991). Relation of the reticuloendothelial function to endotoxin hepatotoxicity. *Exp Pathol*, **43**, 173–179.

182. Dunn, C.W. and Horton, J.W. (1990). Muramyl dipeptide improves mononuclear phagocyte system function in obstructive jaundice. *J Surg Res.*, **48**, 249–253.

183. Kennedy, J.A., McCrory, D.C., Kirk, S.J. and Rowlands, B.J. (1995). L-arginine enhances Kupffer cell secretory responses to exogenous endotoxin in obstructive jaundice. *Br J Surg.*, **82**, 707.

184. Fumarulo, R., Conese, M., Riccardi, S., Giodano, D., Montemurro, P., Colucci, M. and Semeraro, N. (1991). Retinoids inhibit the respiratory burst and degranulation of stimulated human polymorphonuclear leukocytes. *Agents Action*, **34**, 339–344.

185. Conese, M., Montemurro, P., Fumarulo, R., Giodano, D., Riccardi, S., Colucci, M. and Semeraro, N. (1991). Inhibitory effect of retinoids on the generation of procoagulant activity by blood mononuclear phagocytes. *Thrombosis Haemostasis*, **66**, 662–665.

186. Uchino, R., Saitoh, N., Hiraoka, T. and Miyauchi, Y. (1991). Endotoxin-induced lung hemorrhages in obstructive jaundiced rats. *Jap J Surg.*, **21**, 38–42.

187. Uchino, R., Saitoh, N., Hiraoka, T. and Miyauchi, Y. (1992). Effect of activated protein C on impaired fibrinolysis in rats with obstructive jaundice. *Eur Surg Res.*, **24**, 298–301.

188. Puntis, M.C.A. and Jiang, W.G. (1994). Effects of gamma linolenate on human monocyte and hepatoma cells. In "New approaches to cancer treatment: unsaturated lipids and photodynamic therapy" edited by Horrobin D.F. by Churchill-livingstone, pp. 40–67.

*HPB Surgery*, 1997, Vol.10, pp.143–147
Reprints available directly from the publisher
Photocopying permitted by license only

© 1997 OPA (Overseas Publishers Association)
Amsterdam B.V. Published in The Netherlands
by Harwood Academic Publishers
Printed in India

### The Internal Biliary Fistula – Reappraisal of Incidence, Type, Diagnosis and Management of 33 Consecutive Cases

HIROYUKI YAMASHITA, KAZUO CHIJIIWA, YOSHIAKI OGAWA, SYOJI KUROKI and MASAO TANAKA

Department of Surgery I, Kyushu University Faculty of Medicine, Fukuoka, Japan

*(Received 20 June 1994)*

To reevaluate the current features of spontaneous internal biliary fistulas, we reviewed 1,929 consecutive patients who had been treated for biliary tract diseases during the recent 12-year period. Thirty-three patients had internal biliary fistulas and the incidence was 1.9%. Of 33 patients, 20 were women and 13 were men with the average age 63 years, and their mean duration of illness was 4 years. A total of 37 fistulas were found and the most common type was choledochoduodenal (62%), followed by cholecystoduodenal (19%), cholecystocholedochal (11%) and cholecystocolonic (8%) fistulas. Internal biliary fistulas of thirty-one patients were caused by biliary stones and those of two patients by malignant tumors. All of the 17 bile samples examined were bacteria positive and the majority of calculi were brown pigment stones. All of the choledochoduodenal fistulas were correctly diagnosed by endoscopic retrograde cholangiography. In 14 patients with cholecystoenteric or cholecystocholedochal fistulas, direct evidence of the internal fistula was obtained only in 7 patients (50%) preoperatively. Pneumobilia, a small atrophic gallbladder adherent to the neighboring organs and a history of spontaneous disappearance of jaundice in elderly patients may indicate the presence of a cholecystoentric fistula. Since the preoperative diagnostic rate for internal biliary fistula involving the gallbladder is still low, care is necessary before and at the time of surgery especially during laparoscopic cholecystectomy for elderly patients with cholelithiasis.

KEY WORDS: Internal biliary fistula cholelithiasisendoscopicretrogradecholangiography laparoscopic cholecystectomy

### INTRODUCTION

Although traditional open cholecystectomy and recently developed laparoscopic cholecystectomy show very low mortality and morbidity rates[1,2], the presence of spontaneous internal biliary fistulae is reported to increase the morbidity rate considerably and its potential lethality has been appreciated[3–5]. The reported incidence of internal biliary fistulas is about 2% of total biliary diseases and the most common type has been reported to be a cholecystoduodenal fistula, followed by cholecystocolonic and cholecystogastric fistulas[3,4]. Their clinical importance as well as the management depends on the etiology and type of the fistulas. The treatment option can be selected only when such information is available. Although it is sometimes difficult to diagnose correctly the type of internal biliary fistula preoperatively, surgeons must be aware of the possible presence of an internal biliary fistula before surgical intervention. This might be particularly true when considering laparoscopic cholecystectomy.

The recent advances in hepatobiliary imaging techniques[6] have allowed us to reevaluate hepatobiliary diseases. With the aid of endoscopic retrograde cholangiography, choledochoduodenal fistulas in the periampullary region have been found more frequently than ever[7–9]. Despite the improvement in imaging modalities, little literature is currently available about

*All correspondence and reprints requests should be addressed to:* Hiroyuki Yamshita, M.D. Department of Surgery I, Kyushu University Faculty of Medicine 3-1-1 Maidashi, Higashi-ku, Fukuoka 812-82, Japan. Tel: 81-92-641-1151 Ext. 2394, Fax: 81-92-632-2478.

